# Supplementary material for: Variant-specific antibody correlates of protection against SARS-CoV-2 Omicron symptomatic and overall infections
Source: Nat Commun. 2025 Nov 21;16:10305. doi: 10.1038/s41467-025-65235-8 (PMC12639126; doi:10.1038/s41467-025-65235-8)
Supplement: Supplementary file 1 — Supplementary Information [file 41467_2025_65235_MOESM1_ESM.pdf]

Supplementary material for

Variant-specific antibody correlates of protection against SARS-CoV-2 Omicron symptomatic and overall infections

Jose Victor Zambrana[1,2*], Ian A. Mellis[3,4,5,*], Abigail Shotwell[1], Hannah E. Maier[1], Yara Saborio[2,6], Carlos Barillas[2], Roger Lopez[2,6], Gerald Vasquez[2], Miguel Plazaola[2], Nery Sanchez[2], Sergio Ojeda[2], Isabel Gilbertson[1], Guillermina Kuan[2,7], Qian Wang[3], Lihong Liu[3], Angel Balmaseda[2,6], David D. Ho[3#], Aubree Gordon[1#]

[1]Department of Epidemiology, University of Michigan, Ann Arbor, MI
[2]Sustainable Sciences Institute, Managua, Nicaragua
[3]Aaron Diamond AIDS Research Center, Vagelos College of Physicians and Surgeons, Columbia University Irving Medical Center, New York, NY
[4]Department of Pathology and Cell Biology, Vagelos College of Physicians and Surgeons, Columbia University Irving Medical Center, New York, NY
[5]New York Blood Center, New York, NY
[6]Laboratorio Nacional de Virología, Centro Nacional de Diagnóstico y Referencia, Ministerio de Salud, Managua, Nicaragua
[7]Centro de Salud Sócrates Flores Vivas, Ministerio de Salud, Managua, Nicaragua

*Jose Victor Zambrana and Ian A. Mellis contributed equally to this manuscript.
#Correspondence: Aubree Gordon. Department of Epidemiology, University of Michigan, Ann Arbor, 5622 SPH I 1415 Washington Heights Ann Arbor, MI 48109, gordonal@umich.edu (gordonal@umich.edu). #Alternate Correspondence: David D. Ho, Aaron Diamond AIDS Research Center, Columbia University Irving Medical Center, 701 West 168th Street, HHSC 1102, New York, NY 10032, dh2994@cumc.columbia.edu

Supplementary methods

Infection histories

We employed positive serology screens, antibody titer measurements (including four-fold increases), and RT-PCR positivity to identify primary and secondary infections. To qualify as an infection, serological samples had to be from participants aged at least 6 months to avoid misclassification of maternal antibodies. First infections confirmed by RBD screening or spike titer were required to precede the individual's initial COVID-19 vaccination. Serologically distinct infections were defined as those occurring ≥6 months apart, and secondary infections detected via spike titer could not include samples collected around the time of vaccination. For RT-PCR-based detection, infections had to be ≥60 days apart, and sequential infections identified by RT-PCR and a four-fold increase in titer had to be >90 days apart. Any infections identified within a 60-day window were flagged for manual review. For this study, we categorized infection histories by any prior infection vs natural infection naïve. Only RT-PCR-confirmed infections were used to evaluate BA.1 or BA.2 infections.

Laboratory methods

Cell lines. Vero-E6 (CRL-1586) cells and HEK293T (CRL-3216) cells were obtained from ATCC and cultured at 37°C plus 5% CO_2 in Dulbecco's Modified Eagle Medium (DMEM, Sigma) + 10% Fetal Bovine Serum (FBS, Sigma) + 1% penicillin-streptomycin (Sigma). Vero-E6 cells are derived from African green monkey kidneys. HEK293T cells are female human in origin. Cell line morphology of 293T and Vero-E6 was visually confirmed with each passage.

Pseudovirus production. VSV-based SARS-CoV-2 pseudoviruses, in which the native VSV glycoprotein was replaced by SARS-CoV-2 spike and its variants, were produced as previously described. Plasmids containing the appropriate spike were transfected into HEK293T cells with PEI. 24 hours later, VSV-G pseudotyped ΔG-luciferase (G*ΔG-luciferase, Kerafast) was added to the cells, and then washed with culture medium three times before being cultured in fresh medium for another 24 hours. Anti-VSVG (I1) antibody was added to deplete non-pseudotyped viruses. Pseudoviruses were then harvested, centrifuged, aliquoted, and stored at -80°C.

Pseudovirus titration. All serum samples were heat inactivated at 56°C for 30 min before use. Pseudovirus particles bearing various SARS-CoV-2 spike proteins were inoculated onto Vero-E6 cells, starting with 50 µl per well in 96-well plates and then subjected to serial dilutions. Following a 16–18-hour incubation at 37°C, the activity of the virus-encoded firefly luciferase in cell lysates was measured using a SpectraMax luminometer. Each SARS-CoV-2 pseudovirus was titrated to standardize viral infectious dose before use in neutralization assays. The 50% tissue culture infectious dose ($TCID_{50}$) was calculated for each pseudovirus and used in all subsequent neutralization experiments.

Pseudovirus serum neutralization assays. Serially diluted (seven dilutions of) heat-inactivated sera were added in 96-well plates, starting at 1:100 dilution. Pseudoviruses were added and incubated at 37 °C for 1 hour. In each plate, wells containing only pseudoviruses were included as controls. Vero-E6 cells were then added at a density of 4×10^4 cells per well and incubate at 37 °C for an additional 16 hours. Cells were lysed and luminescence was determined by the Luciferase Assay System (Promega) and SoftMax Pro v.7.0.2 (Molecular Devices) according to the manufacturers' instructions. Data were analyzed in R v4.3.2, and ID50s were calculated using a 5-parameter log-logistic model with drda v2.0.3.

Supplementary tables

Table S1. Univariate analysis by Infection, assay and Omicron wave.

Omicron wave	Assay	Median difference*	statistic	P-value
BA.1 wave	BA.1 Neutralization, $Log_4[ID_{50}]$	0.32 (-0.03, 0.75)	8455.0	0.0878
BA.2 wave	BA.1 Neutralization, $Log_4[ID_{50}]$	0.83 (0.36, 1.42)	1549.0	0.000427
BA.1 wave	BA.2 Neutralization, $Log_4[ID_{50}]$	0.18 (-0.11, 0.56)	8140.0	0.252
BA.2 wave	BA.2 Neutralization, $Log_4[ID_{50}]$	0.87 (0.34, 1.47)	1539.0	0.000567
BA.1 wave	D614G Neutralization, $Log_4[ID_{50}]$	0.21 (-0.14, 0.55)	8157.5	0.240
BA.2 wave	D614G Neutralization, $Log_4[ID_{50}]$	0.69 (0.24, 1.16)	1481.0	0.00266
BA.1 wave	Spike Binding, $Log_4[Titer]$	0 (-0.35, 0.34)	7491.0	0.939
BA.2 wave	Spike Binding, $Log_4[Titer]$	0.49 (0.02, 1.01)	1362.5	0.0357

*Estimate and 95% confidence interval

Table S2. Univariate analysis by symptomatic infections, assay and Omicron wave.

Omicron wave	Assay	Median difference*	statistic	P-value
BA.1 wave	BA.1 Neutralization, $Log_4[ID_{50}]$	0.6 (0.12, 1.05)	8023.0	0.00937
BA.2 wave	BA.1 Neutralization, $Log_4[ID_{50}]$	1.02 (0.41, 1.79)	1364.5	0.000559
BA.1 wave	BA.2 Neutralization, $Log_4[ID_{50}]$	0.38 (0, 0.81)	7726.0	0.0417
BA.2 wave	BA.2 Neutralization, $Log_4[ID_{50}]$	0.98 (0.37, 1.64)	1339.0	0.00119
BA.1 wave	D614G Neutralization, $Log_4[ID_{50}]$	0.3 (-0.07, 0.67)	7505.5	0.106
BA.2 wave	D614G Neutralization, $Log_4[ID_{50}]$	0.79 (0.27, 1.32)	1299.0	0.00357
BA.1 wave	Spike Binding, $Log_4[Titer]$	0.21 (-0.08, 0.64)	7250	0.189
BA.2 wave	Spike Binding, $Log_4[Titer]$	0.67 (0.13, 1.17)	1245.5	0.0133

*Estimate and 95% confidence interval

Table S3 - Multivariate analysis by infection status, Omicron wave and assay.

Assay	Omicron wave	Variable	OR (95% CI)	Protection% (95% CI)	Standard error	Z statistic	P-value	Residual df
BA.1 Neutralization, $Log_4[ID_{50}]$	BA.1 wave	Titer	0.88 (0.73, 1.05)	12 (-5, 27)	0.094	-1.407	0.160	246
		Prior Vaccination	1.53 (0.85, 2.77)	-53 (-177, 15)	0.302	1.405	0.160	
		Prior infection	0.31 (0.07, 1.03)	69 (-3, 93)	0.677	-1.748	0.0804	
		Age	1.02 (1, 1.03)	-2 (-3, 0)	0.008	2.302	0.0213	
	BA.2 wave	**Titer**	**0.55 (0.36, 0.79)**	**45 (21, 64)**	**0.196**	**-3.029**	**0.00246**	**89**
		Prior Vaccination	0.98 (0.34, 2.91)	2 (-191, 66)	0.541	-0.044	0.965	
		Prior infection	0.31 (0.01, 2.52)	69 (-152, 99)	1.189	-0.982	0.326	
		Age	1 (0.98, 1.02)	0 (-2, 2)	0.010	-0.163	0.870	
BA.2 Neutralization, $Log_4[ID_{50}]$	BA.1 wave	Titer	0.87 (0.72, 1.06)	13 (-6, 28)	0.099	-1.375	0.169	246
		Prior Vaccination	1.56 (0.85, 2.88)	-56 (-188, 15)	0.309	1.445	0.149	
		Prior infection	0.31 (0.07, 1.06)	69 (-6, 93)	0.680	-1.712	0.0869	
		age	1.02 (1, 1.03)	-2 (-3, 0)	0.007	2.402	0.0163	
	BA.2 wave	**Titer**	**0.55 (0.36, 0.79)**	**45 (21, 64)**	**0.198**	**-2.987**	**0.00281**	**89**
		Prior Vaccination	1 (0.35, 2.98)	0 (-198, 65)	0.542	-0.003	0.998	
		Prior infection	0.33 (0.02, 2.98)	67 (-198, 98)	1.226	-0.905	0.365	
		Age	1 (0.98, 1.02)	0 (-2, 2)	0.010	0.032	0.974	
D614G Neutralization, $Log_4[ID_{50}]$	BA.1 wave	Titer	0.87 (0.69, 1.09)	13 (-9, 31)	0.115	-1.228	0.219	246
		Prior Vaccination	1.56 (0.84, 2.9)	-56 (-190, 16)	0.314	1.413	0.158	
		Prior infection	0.3 (0.07, 1.01)	70 (-1, 93)	0.676	-1.777	0.0755	
		Age	1.02 (1, 1.03)	-2 (-3, 0)	0.007	2.430	0.0151	
	BA.2 wave	**Titer**	**0.49 (0.29, 0.77)**	**51 (23, 71)**	**0.246**	**-2.892**	**0.00383**	**89**
		Prior Vaccination	1.38 (0.46, 4.45)	-38 (-345, 54)	0.574	0.562	0.574	
		Prior infection	0.24 (0.01, 1.83)	76 (-83, 99)	1.173	-1.225	0.221	
		Age	1 (0.98, 1.02)	0 (-2, 2)	0.010	0.155	0.877	
Spike Binding, $Log_4[Titer]$	BA.1 wave	Titer	1.01 (0.85, 1.2)	-1 (-20, 15)	0.088	0.077	0.938	245
		Prior Vaccination	1.28 (0.71, 2.33)	-28 (-133, 29)	0.304	0.821	0.412	
		Prior infection	0.24 (0.05, 0.83)	76 (17, 95)	0.694	-2.067	0.0388	
		Age	1.02 (1, 1.03)	-2 (-3, 0)	0.007	2.537	0.0112	
	BA.2 wave	Titer	0.7 (0.45, 1.02)	30 (-2, 55)	0.204	-1.776	0.0758	89
		Prior Vaccination	1.04 (0.36, 3.13)	-4 (-213, 64)	0.547	0.080	0.936	
		Prior infection	0.18 (0.01, 1.29)	82 (-29, 99)	1.147	-1.507	0.132	
		Age	1 (0.98, 1.02)	0 (-2, 2)	0.010	0.036	0.971	

Table S4. Multivariate analysis by symptomatic infection status, Omicron wave and assay.

Assay	Omicron wave	Variable	OR (95% CI)	Protection (95% CI)	Standard error	Z statistic	P-value	Residual df
BA.1 Neutralization, $Log_4[ID_{50}]$	BA.1 wave	**Titer**	**0.72 (0.58, 0.88)**	**28 (12, 42)**	**0.104**	**-3.148**	**0.00164**	246
		Prior Vaccination	1.57 (0.82, 3.1)	-57 (-210, 18)	0.338	1.338	0.181	
		Prior infection	3.9 (1.11, 18.6)	-290 (-1760, -11)	0.700	1.944	0.0519	
		age	1.02 (1, 1.03)	-2 (-3, 0)	0.007	2.453	0.0142	
	BA.2 wave	**Titer**	**0.53 (0.35, 0.76)**	**47 (24, 65)**	**0.190**	**-3.321**	**0.000897**	**89**
		Prior Vaccination	2.11 (0.65, 8.29)	-111 (-729, 35)	0.641	1.166	0.244	
		Prior infection	0.5 (0.05, 4)	50 (-300, 95)	1.071	-0.647	0.518	
		age	0.99 (0.96, 1.01)	1 (-1, 4)	0.012	-0.971	0.331	
BA.2 Neutralization, $Log_4[ID_{50}]$	BA.1 wave	**Titer**	**0.72 (0.58, 0.89)**	**28 (11, 42)**	**0.108**	**-2.997**	**0.00273**	246
		Prior Vaccination	1.65 (0.84, 3.31)	-65 (-231, 16)	0.347	1.433	0.152	
		Prior infection	3.93 (1.11, 18.95)	-293 (-1795, -11)	0.705	1.941	0.0522	
		age	1.02 (1.01, 1.03)	-2 (-3, -1)	0.007	2.658	0.00786	
	BA.2 wave	**Titer**	**0.57 (0.38, 0.8)**	**43 (20, 62)**	**0.186**	**-3.061**	**0.00220**	**89**
		Prior Vaccination	2.04 (0.63, 7.87)	-104 (-687, 37)	0.633	1.125	0.261	
		Prior infection	0.55 (0.05, 5.11)	45 (-411, 95)	1.122	-0.532	0.595	
		age	0.99 (0.97, 1.01)	1 (-1, 3)	0.011	-0.746	0.455	
D614G Neutralization, $Log_4[ID_{50}]$	BA.1 wave	**Titer**	**0.72 (0.56, 0.93)**	**28 (7, 44)**	**0.130**	**-2.504**	**0.0123**	246
		Prior Vaccination	1.58 (0.8, 3.21)	-58 (-221, 20)	0.351	1.308	0.191	
		Prior infection	3.4 (0.97, 16.22)	-240 (-1522, 3)	0.698	1.753	0.0795	
		age	1.02 (1.01, 1.03)	-2 (-3, -1)	0.007	2.738	0.00618	
	BA.2 wave	**Titer**	**0.43 (0.25, 0.71)**	**57 (29, 75)**	**0.264**	**-3.161**	**0.00157**	**89**
		Prior Vaccination	3.41 (0.94, 15.8)	-241 (-1480, 6)	0.709	1.728	0.0840	
		Prior infection	0.39 (0.04, 3.05)	61 (-205, 96)	1.066	-0.890	0.373	
		age	0.99 (0.97, 1.02)	1 (-2, 3)	0.012	-0.625	0.532	
Spike Binding, $Log_4[Titer]$	BA.1 wave	**Titer**	**0.82 (0.67, 0.99)**	**18 (1, 33)**	**0.098**	**-2.074**	**0.0381**	245
		Prior Vaccination	1.37 (0.71, 2.69)	-37 (-169, 29)	0.338	0.929	0.353	
		Prior infection	3.34 (0.92, 16.48)	-234 (-1548, 8)	0.717	1.684	0.0922	
		age	1.02 (1.01, 1.04)	-2 (-4, -1)	0.007	3.149	0.00164	
	BA.2 wave	**Titer**	**0.58 (0.36, 0.87)**	**42 (13, 64)**	**0.220**	**-2.481**	**0.0131**	89
		Prior Vaccination	2.51 (0.75, 10.14)	-151 (-914, 25)	0.653	1.412	0.158	
		Prior infection	0.27 (0.03, 1.93)	73 (-93, 97)	1.018	-1.290	0.197	
		age	0.99 (0.97, 1.02)	1 (-2, 3)	0.011	-0.543	0.587	

Table S5. Multivariate analysis by infection status, Omicron wave and assay, robust confidence intervals. Adjusted for household ID. Corr. Para. = Correlation parameter. Resid. Df. = residual degrees of freedom.

Assay	Omicron wave	Variable	OR (95% CI)	Protection% (95% CI)	SE	Z statistic	P-value	Corr. Para.	Resid. df
BA.1 Neutralization, $Log_4[ID_{50}]$	BA.1 wave	Titer	0.92 (0.77, 1.09)	8 (-9, 23)	0.088	0.996	0.318	0.153	246
		Prior Vaccination	1.51 (0.81, 2.82)	-51 (-182, 19)	0.320	1.655	0.198		
		Prior infection	0.31 (0.1, 0.95)	69 (5, 90)	0.574	4.198	0.0405		
		Age	1.02 (1, 1.03)	-2 (-3, 0)	0.007	6.455	0.0111		
	BA.2 wave	**Titer**	**0.55 (0.39, 0.76)**	**45 (24, 61)**	**0.169**	**12.897**	**0.000329**	0.088	89
		Prior Vaccination	0.89 (0.27, 2.89)	11 (-189, 73)	0.601	0.037	0.848		
		Prior infection	0.34 (0.02, 4.69)	66 (-369, 98)	1.336	0.646	0.421		
		Age	1 (0.98, 1.02)	0 (-2, 2)	0.011	0.053	0.819		
BA.2 Neutralization, $Log_4[ID_{50}]$	BA.1 wave	Titer	0.89 (0.75, 1.06)	11 (-6, 25)	0.089	1.778	0.182	0.100	246
		Prior Vaccination	1.59 (0.84, 3.02)	-59 (-202, 16)	0.328	2.005	0.157		
		Prior infection	0.33 (0.11, 1.04)	67 (-4, 89)	0.586	3.559	0.0592		
		age	1.02 (1, 1.03)	-2 (-3, 0)	0.007	6.635	0.0100		
	BA.2 wave	**Titer**	**0.54 (0.39, 0.75)**	**46 (25, 61)**	0.170	13.144	**0.000289**	0.156	89
		Prior Vaccination	0.9 (0.26, 3.1)	10 (-210, 74)	0.632	0.029	0.865		
		Prior infection	0.38 (0.03, 5.47)	62 (-447, 97)	1.360	0.505	0.477		
		Age	1 (0.98, 1.02)	0 (-2, 2)	0.011	0.002	0.967		
D614G Neutralization, $Log_4[ID_{50}]$	BA.1 wave	Titer	0.89 (0.73, 1.08)	11 (-8, 27)	0.100	1.354	0.245	246	246
		Prior Vaccination	1.57 (0.84, 2.95)	-57 (-195, 16)	0.320	2.010	0.156		
		Prior infection	0.32 (0.1, 0.99)	68 (1, 90)	0.579	3.929	0.0475		
		Age	1.02 (1, 1.03)	-2 (-3, 0)	0.007	6.929	0.00848		
	BA.2 wave	**Titer**	**0.5 (0.31, 0.79)**	**50 (21, 69)**	0.237	8.751	**0.00309**	0.086	89
		Prior Vaccination	1.21 (0.37, 3.99)	-21 (-299, 63)	0.609	0.096	0.757		
		Prior infection	0.28 (0.02, 3.11)	72 (-211, 98)	1.231	1.078	0.299		
		Age	1 (0.98, 1.02)	0 (-2, 2)	0.012	0.004	0.952		
Spike Binding, $Log_4[Titer]$	BA.1 wave	Titer	1.01 (0.86, 1.17)	-1 (-17, 14)	0.078	0.009	0.924	0.161	245
		Prior Vaccination	1.34 (0.72, 2.49)	-34 (-149, 28)	0.314	0.874	0.350		
		Prior infection	0.26 (0.09, 0.8)	74 (20, 91)	0.565	5.540	0.0186		
		Age	1.02 (1.01, 1.03)	-2 (-3, -1)	0.007	7.727	0.00544		
	BA.2 wave	Titer	0.71 (0.44, 1.15)	29 (-15, 56)	0.246	1.977	0.160	0.093	89
		Prior Vaccination	0.91 (0.27, 3.09)	9 (-209, 73)	0.624	0.024	0.878		
		Prior infection	0.21 (0.02, 2.14)	79 (-114, 98)	1.174	1.717	0.190		
		Age	1 (0.98, 1.02)	0 (-2, 2)	0.012	0.002	0.966		

Table S6. Multivariate analysis by symptomatic infection status, Omicron wave and assay, robust confidence intervals. Adjusted for household ID. Corr. Para. = Correlation parameter. Resid. Df. = residual degrees of freedom.

Assay	Omicron wave	Variable	OR (95% CI)	Protection% (95% CI)	Standard error	Z statistic	P-value	Corr. Para.	Resid. df
BA.1 Neutralization, Log$_4$[ID$_{50}$]	BA.1 wave	**Titer**	**0.73 (0.6, 0.88)**	**27 (12, 40)**	0.097	10.520	**0.00118**	0.073	246
		Prior Vaccination	1.47 (0.77, 2.83)	-47 (-183, 23)	0.333	1.346	0.246		
		Prior infection	3.58 (0.75, 17.09)	-258 (-1609, 25)	0.797	2.563	0.109		
		Age	1.02 (1, 1.03)	-2 (-3, 0)	0.007	7.214	0.00724		
	BA.2 wave	**Titer**	**0.54 (0.41, 0.71)**	**46 (29, 59)**	0.138	19.440	**0.0000104**	0.095	89
		Prior Vaccination	1.86 (0.55, 6.26)	-86 (-526, 45)	0.620	0.998	0.318		
		Prior infection	0.51 (0.09, 2.78)	49 (-178, 91)	0.864	0.604	0.437		
		Age	0.99 (0.96, 1.01)	1 (-1, 4)	0.013	0.871	0.351		
BA.2 Neutralization, Log$_4$[ID$_{50}$]	BA.1 wave	**Titer**	**0.72 (0.59, 0.89)**	**28 (11, 41)**	0.105	9.604	**0.00194**	0.090	246
		Prior Vaccination	1.56 (0.8, 3.06)	-56 (-206, 20)	0.344	1.672	0.196		
		Prior infection	3.69 (0.77, 17.75)	-269 (-1675, 23)	0.801	2.657	0.103		
		age	1.02 (1.01, 1.03)	-2 (-3, -1)	0.007	7.875	0.00501		
	BA.2 wave	Titer	0.58 (0.44, 0.76)	42 (24, 56)	0.143	14.756	0.000122	0.121	89
		Prior Vaccination	1.72 (0.5, 5.87)	-72 (-487, 50)	0.626	0.749	0.387		
		Prior infection	0.6 (0.12, 2.94)	40 (-194, 88)	0.814	0.405	0.525		
		Age	0.99 (0.97, 1.01)	1 (-1, 3)	0.012	0.591	0.442		
D614G Neutralization, Log$_4$[ID$_{50}$]	BA.1 wave	**Titer**	**0.72 (0.57, 0.9)**	**28 (10, 43)**	0.115	8.315	**0.00393**	0.087	246
		Prior Vaccination	1.52 (0.78, 2.94)	-52 (-194, 22)	0.337	1.528	0.216		
		Prior infection	3.26 (0.65, 16.23)	-226 (-1523, 35)	0.820	2.074	0.150		
		Age	1.02 (1.01, 1.03)	-2 (-3, -1)	0.006	9.338	0.00224		
	BA.2 wave	**Titer**	**0.45 (0.26, 0.78)**	**55 (22, 74)**	0.274	8.307	**0.00395**	0.121	89
		Prior Vaccination	2.68 (0.82, 8.73)	-168 (-773, 18)	0.603	2.672	0.102		
		Prior infection	0.44 (0.09, 2.13)	56 (-113, 91)	0.807	1.042	0.307		
		Age	0.99 (0.97, 1.02)	1 (-2, 3)	0.013	0.353	0.552		
Spike Binding, Log$_4$[Titer]	BA.1 wave	**Titer**	**0.81 (0.68, 0.95)**	**19 (5, 32)**	0.085	6.277	**0.0122**	0.085	245
		Prior Vaccination	1.34 (0.69, 2.6)	-34 (-160, 31)	0.339	0.737	0.391		
		Prior infection	3.27 (0.6, 17.97)	-227 (-1697, 40)	0.869	1.859	0.173		
		Age	1.02 (1.01, 1.04)	-2 (-4, -1)	0.006	14.019	0.000181		
	BA.2 wave	**Titer**	**0.6 (0.37, 0.97)**	**40 (3, 63)**	0.246	4.331	**0.0374**	0.124	89
		Prior Vaccination	2.01 (0.61, 6.56)	-101 (-556, 39)	0.605	1.328	0.249		
		Prior infection	0.31 (0.06, 1.64)	69 (-64, 94)	0.844	1.883	0.170		
		Age	0.99 (0.97, 1.02)	1 (-2, 3)	0.012	0.311	0.577		

Table S7. Multivariate analysis by infection status, Omicron wave and assay, robust confidence intervals. Adjusted for household ID and time since last exposure adjusted. Corr. Para. = Correlation parameter. Resid. Df. = residual degrees of freedom.

Assay	Omicron wave	Variable	OR (95% CI)	Protection% (95% CI)	Standard error	Z statistic	P-value	Corr. Para.	Resid. df
BA.1 Neutralization, $Log_4[ID_{50}]$	BA.1 wave	Titer	0.92 (0.76, 1.1)	8 (-10, 24)	0.094	0.851	0.356	0.144	237
		Prior Vaccination	1.12 (0.41, 3.04)	-12 (-204, 59)	0.512	0.045	0.831		
		Prior infection	0.26 (0.05, 1.45)	74 (-45, 95)	0.883	2.369	0.124		
		Age	1.02 (1, 1.03)	-2 (-3, 0)	0.007	6.291	0.0121		
		Time since last exposure	1 (1, 1)	0 (0, 0)	0.001	1.094	0.296		
	BA.2 wave	**Titer**	**0.52 (0.33, 0.81)**	**48 (19, 67)**	**0.227**	**8.258**	**0.00406**	0.037	87
		Prior Vaccination	0.42 (0.08, 2.22)	58 (-122, 92)	0.844	1.030	0.310		
		Prior infection	0.74 (0.05, 12.11)	26 (-1111, 95)	1.427	0.045	0.832		
		Age	0.99 (0.97, 1.02)	1 (-2, 3)	0.012	0.298	0.585		
		Time since last exposure	1 (0.99, 1)	0 (0, 1)	0.003	2.364	0.124		
BA.2 Neutralization, $Log_4[ID_{50}]$	BA.1 wave	Titer	0.89 (0.74, 1.08)	11 (-8, 26)	0.098	1.368	0.242	0.149	237
		Prior Vaccination	1.2 (0.43, 3.31)	-20 (-231, 57)	0.519	0.120	0.729		
		Prior infection	0.27 (0.05, 1.53)	73 (-53, 95)	0.888	2.196	0.138		
		Age	1.02 (1, 1.03)	-2 (-3, 0)	0.007	6.414	0.0113		
		Time since last exposure	1 (1, 1)	0 (0, 0)	0.001	0.928	0.335		
	BA.2 wave	**Titer**	**0.52 (0.33, 0.83)**	**48 (17, 67)**	**0.232**	**7.729**	**0.00543**	0.052	87
		Prior Vaccination	0.47 (0.09, 2.5)	53 (-150, 91)	0.856	0.794	0.373		
		Prior infection	0.86 (0.05, 14.38)	14 (-1338, 95)	1.438	0.011	0.916		
		Age	1 (0.97, 1.02)	0 (-2, 3)	0.011	0.144	0.705		
		Time since last exposure	1 (0.99, 1)	0 (0, 1)	0.003	1.731	0.188		
D614G Neutralization, $Log_4[ID_{50}]$	BA.1 wave	Titer	0.89 (0.72, 1.09)	11 (-9, 28)	0.105	1.352	0.245	0.146	237
		Prior Vaccination	1.18 (0.43, 3.22)	-18 (-222, 57)	0.511	0.107	0.744		
		Prior infection	0.25 (0.05, 1.42)	75 (-42, 95)	0.880	2.431	0.119		
		Age	1.02 (1, 1.03)	-2 (-3, 0)	0.007	6.725	0.00951		
		Time since last exposure	1 (1, 1)	0 (0, 0)	0.001	1.026	0.311		
	BA.2 wave	**Titer**	**0.51 (0.29, 0.88)**	**49 (12, 71)**	**0.280**	**5.901**	**0.0151**	0.039	87
		Prior Vaccination	0.68 (0.15, 3.19)	32 (-219, 85)	0.788	0.239	0.625		
		Prior infection	0.49 (0.04, 6.26)	51 (-526, 96)	1.299	0.300	0.584		
		Age	1 (0.97, 1.02)	0 (-2, 3)	0.012	0.067	0.795		
		Time since last exposure	1 (0.99, 1)	0 (0, 1)	0.003	1.269	0.260		
Spike Binding, $Log_4[Titer]$	BA.1 wave	Titer	1.02 (0.86, 1.21)	-2 (-21, 14)	0.087	0.055	0.815	0.153	236
		Prior Vaccination	0.91 (0.33, 2.51)	9 (-151, 67)	0.518	0.033	0.857		
		Prior infection	0.21 (0.04, 1.18)	79 (-18, 96)	0.871	3.125	0.0771		
		Age	1.02 (1.01, 1.03)	-2 (-3, -1)	0.007	7.501	0.00617		
		Time since last exposure	1 (1, 1)	0 (0, 0)	0.001	1.581	0.209		
	BA.2 wave	**Titer**	**0.75 (0.42, 1.33)**	**25 (-33, 58)**	**0.296**	**0.978**	**0.323**	0.038	87
		Prior Vaccination	0.43 (0.08, 2.27)	57 (-127, 92)	0.847	0.986	0.321		
		Prior infection	0.31 (0.02, 4.1)	69 (-310, 98)	1.310	0.781	0.377		
		Age	1 (0.97, 1.02)	0 (-2, 3)	0.011	0.119	0.730		
		Time since last exposure	1 (0.99, 1)	0 (0, 1)	0.003	1.584	0.208		

Table S8. Multivariate analysis by symptomatic infection status, Omicron wave and assay, robust confidence intervals. Adjusted for household ID and time since last exposure adjusted. Corr. Para. = Correlation parameter. Resid. Df. = residual degrees of freedom.

Assay	Omicron wave	Variable	OR (95% CI)	Protection% (95% CI)	Standard error	Z statistic	P-value	Corr. Para.	Resid. df
BA.1 Neutralization, $Log_4[ID_{50}]$	BA.1 wave	**Titer**	**0.78 (0.65, 0.94)**	**22 (6, 35)**	**0.095**	**6.727**	**0.00950**	0.098	237
		Prior Vaccination	0.62 (0.23, 1.64)	38 (-64, 77)	0.497	0.928	0.335		
		Prior infection	2.75 (0.3, 25.44)	-175 (-2444, 70)	1.134	0.797	0.372		
		Age	1.02 (1.01, 1.04)	-2 (-4, -1)	0.007	10.018	0.00155		
		Time since last exposure	1 (0.99, 1)	0 (0, 1)	0.001	6.766	0.00929		
	BA.2 wave	**Titer**	**0.56 (0.42, 0.75)**	**44 (25, 58)**	**0.144**	**15.943**	**0.0000653**	0.079	87
		Prior Vaccination	1.21 (0.27, 5.35)	-21 (-435, 73)	0.760	0.062	0.804		
		Prior infection	0.39 (0.05, 3.33)	61 (-233, 95)	1.093	0.738	0.390		
		Age	0.99 (0.96, 1.01)	1 (-1, 4)	0.014	0.843	0.358		
		Time since last exposure	1 (0.99, 1)	0 (0, 1)	0.002	0.854	0.355		
BA.2 Neutralization, $Log_4[ID_{50}]$	BA.1 wave	Titer	**0.78 (0.63, 0.95)**	**22 (5, 37)**	**0.103**	**6.009**	**0.0142**	0.111	237
		Prior Vaccination	0.65 (0.24, 1.73)	35 (-73, 76)	0.501	0.751	0.386		
		Prior infection	2.68 (0.31, 23.29)	-168 (-2229, 69)	1.103	0.800	0.371		
		Age	1.02 (1.01, 1.04)	-2 (-4, -1)	0.007	10.704	0.00107		
		Time since last exposure	1 (0.99, 1)	0 (0, 1)	0.001	6.621	0.0101		
	BA.2 wave	**Titer**	**0.6 (0.45, 0.81)**	**40 (19, 55)**	**0.153**	**11.096**	**0.000865**	0.108	87
		Prior Vaccination	1.21 (0.27, 5.39)	-21 (-439, 73)	0.762	0.063	0.802		
		Prior infection	0.44 (0.06, 3.3)	56 (-230, 94)	1.030	0.640	0.424		
		Age	0.99 (0.97, 1.01)	1 (-1, 3)	0.012	0.603	0.437		
		Time since last exposure	1 (0.99, 1)	0 (0, 1)	0.002	0.439	0.508		
D614G Neutralization, $Log_4[ID_{50}]$	BA.1 wave	**Titer**	**0.77 (0.61, 0.96)**	**23 (4, 39)**	**0.115**	**5.171**	**0.0230**	0.111	237
		Prior Vaccination	0.61 (0.23, 1.62)	39 (-62, 77)	0.497	0.970	0.325		
		Prior infection	2.31 (0.26, 20.5)	-131 (-1950, 74)	1.113	0.567	0.452		
		Age	1.02 (1.01, 1.04)	-2 (-4, -1)	0.007	11.785	0.000597		
		Time since last exposure	1 (0.99, 1)	0 (0, 1)	0.001	7.257	0.00706		
	BA.2 wave	**Titer**	**0.48 (0.27, 0.84)**	**52 (16, 73)**	**0.287**	**6.573**	**0.0104**	0.104	87
		Prior Vaccination	1.95 (0.47, 8.12)	-95 (-712, 53)	0.729	0.834	0.361		
		Prior infection	0.33 (0.05, 2.36)	67 (-136, 95)	1.000	1.216	0.270		
		Age	0.99 (0.97, 1.02)	1 (-2, 3)	0.013	0.360	0.549		
		Time since last exposure	1 (0.99, 1)	0 (0, 1)	0.002	0.266	0.606		
Spike Binding, $Log_4[Titer]$	BA.1 wave	**Titer**	0.85 (0.72, 1.01)	15 (-1, 28)	0.084	3.601	0.0577	0.105	236
		Prior Vaccination	0.52 (0.2, 1.36)	48 (-36, 80)	0.485	1.776	0.183		
		Prior infection	2.18 (0.25, 18.7)	-118 (-1770, 75)	1.096	0.508	0.476		
		Age	1.03 (1.01, 1.04)	-3 (-4, -1)	0.007	15.592	0.0000786		
		Time since last exposure	1 (0.99, 1)	0 (0, 1)	0.001	7.695	0.00554		
	BA.2 wave	Titer	0.64 (0.39, 1.04)	36 (-4, 61)	0.253	3.214	0.0730	0.105	87
		Prior Vaccination	1.35 (0.38, 4.86)	-35 (-386, 62)	0.652	0.216	0.642		
		Prior infection	0.22 (0.03, 1.74)	78 (-74, 97)	1.044	2.046	0.153		
		Age	0.99 (0.97, 1.02)	1 (-2, 3)	0.012	0.334	0.563		
		Time since last exposure	1 (0.99, 1)	0 (0, 1)	0.002	0.422	0.516		

Table S9. Protective thresholds – GAM model

Assay	Omicron wave	50% TS - Infection	80% TS - Infection	50% TS - Symptomatic infection	80% TS - Symptomatic infection
BA.1 Neutralization, ID_{50}	BA.1 Wave	-	-	39	13107
BA.2 Neutralization, ID_{50}		-	-	53	-
D614G Neutralization, ID_{50}		-	-	222	78694
Spike Binding, Titer		-	-	40	-
BA.1 Neutralization, ID_{50}	BA.2 Wave	522	11809	186	3834
BA.2 Neutralization, ID_{50}		588	14685	181	5162
D614G Neutralization, ID_{50}		2276	33036	1145	11962
Spike Binding, Titer		2581	-	1465	48560

TS: Threshold. Protective thresholds to achieve 50% or 80% reduction in risk were derived from multivariate model estimate predictions based on vaccinated adults with prior natural infections.

Table S10. Protective thresholds – GLM model

Assay	Omicron wave	50% TS - Infection	80% TS - Infection	50% TS - Symptomatic infection	80% TS - Symptomatic infection
BA.1 Neutralization, ID_{50}	BA.1 Wave	-	-	39	13107
BA.2 Neutralization, ID_{50}		-	-	53	-
D614G Neutralization, ID_{50}		-	-	222	78694
Spike Binding, Titer		-	-	46	-
BA.1 Neutralization, ID_{50}	BA.2 Wave	506	12540	186	3835
BA.2 Neutralization, ID_{50}		588	14685	181	5163
D614G Neutralization, ID_{50}		2276	33032	1188	11635
Spike Binding, Titer		2581	-	1465	48560

TS: Threshold. Protective thresholds to achieve 50% or 80% reduction in risk were derived from multivariate model estimate predictions based on vaccinated adults with prior natural infections.

.

Table S11. Mediation analyses

Exposure	Mediator	Outcome	Term	RR (95%)	Protection (95% CI)*	P-value
Prior infection	Homotypic Neuts	Infection	Total Effect	0.75 (0.63, 0.89)	25 (37, 11)	0.006
			**ACME (average)**	**0.89 (0.79, 0.96)**	**11 (21, 4)**	**0.002**
			ADE (average)	0.84 (0.69, 1.02)	16 (31, -2)	0.072
		Symptomatic Infection	Total Effect	1.04 (0.82, 1.22)	-4 (18, -22)	0.700
			**ACME (average)**	**0.88 (0.76, 0.94)**	**12 (24, 6)**	**0.002**
			ADE (average)	1.18 (0.99, 1.37)	-18 (1, -37)	0.052
Prior Vax		Infection	Total Effect	1.04 (0.93, 1.15)	-4 (7, -15)	0.460
			**ACME (average)**	**0.95 (0.91, 0.98)**	**5 (9, 2)**	**0.000**
			ADE (average)	1.1 (0.98, 1.22)	-10 (2, -22)	0.110
		Symptomatic Infection	Total Effect	1.04 (0.94, 1.16)	-4 (6, -16)	0.414
			**ACME (average)**	**0.93 (0.89, 0.96)**	**7 (11, 4)**	**0.000**
			ADE (average)	1.12 (1.02, 1.24)	-12 (-2, -24)	0.016
Prior infection	Neut Ancestral	Infection	Total Effect	0.75 (0.63, 0.89)	25 (37, 11)	0.006
			**ACME (average)**	**0.91 (0.82, 0.98)**	**9 (18, 2)**	**0.008**
			ADE (average)	0.82 (0.69, 1)	18 (31, 0)	0.042
		Symptomatic Infection	Total Effect	1.04 (0.82, 1.23)	-4 (18, -23)	0.708
			**ACME (average)**	**0.9 (0.8, 0.95)**	**10 (20, 5)**	**0.000**
			ADE (average)	1.15 (0.95, 1.37)	-15 (5, -37)	0.128
Prior Vax		Infection	Total Effect	1.04 (0.93, 1.15)	-4 (7, -15)	0.462
			**ACME (average)**	**0.93 (0.88, 0.99)**	**7 (12, 1)**	**0.008**
			ADE (average)	1.11 (0.99, 1.26)	-11 (1, -26)	0.078
		Symptomatic Infection	Total Effect	1.04 (0.94, 1.15)	-4 (6, -15)	0.450
			**ACME (average)**	**0.91 (0.85, 0.95)**	**9 (15, 5)**	**0.000**
			ADE (average)	1.14 (1.04, 1.28)	-14 (-4, -28)	0.002
Prior infection	Spike Binding Ancestral	Infection	Total Effect	0.75 (0.64, 0.9)	25 (36, 10)	0.002
			ACME (average)	0.98 (0.88, 1.05)	2 (12, -5)	0.574
			ADE (average)	0.76 (0.64, 0.95)	24 (36, 5)	0.018
		Symptomatic Infection	Total Effect	1.04 (0.82, 1.23)	-4 (18, -23)	0.656
			**ACME (average)**	**0.92 (0.82, 0.98)**	**8 (18, 2)**	**0.006**
			ADE (average)	1.13 (0.91, 1.38)	-13 (9, -38)	0.206
Prior Vax		Infection	Total Effect	1.04 (0.93, 1.15)	-4 (7, -15)	0.514
			ACME (average)	0.99 (0.94, 1.04)	1 (6, -4)	0.574
			ADE (average)	1.05 (0.93, 1.17)	-5 (7, -17)	0.412
		Symptomatic Infection	Total Effect	1.03 (0.93, 1.14)	-3 (7, -14)	0.558
			**ACME (average)**	**0.94 (0.9, 0.98)**	**6 (10, 2)**	**0.006**
			ADE (average)	1.09 (0.98, 1.21)	-9 (2, -21)	0.114

*Protection calculated as (1-RR)x100.

Table S12. GenBank accessions for SARS-CoV-2 sequenced genomes from study-participant samples

<table>
<tr><th>Sequence name</th><th>GenBank Accession ID</th><th>Collection date</th><th>Lineage</th></tr>
<tr><td>hCoV-19/Nicaragua/CNDR-11089-03/2022|EPI_ISL_17065848|2022-01-22</td><td>PX441928</td><td>1/22/2022</td><td>BA.1</td></tr>
<tr><td>hCoV-19/Nicaragua/CNDR-10014-03/2022|EPI_ISL_17065846|2022-01-29</td><td>PX441930</td><td>1/29/2022</td><td>BA.1</td></tr>
<tr><td>hCoV-19/Nicaragua/CNDR-10297-03/2022|EPI_ISL_17065847|2022-01-29</td><td>PX441929</td><td>1/29/2022</td><td>BA.1</td></tr>
<tr><td>hCoV-19/Nicaragua/CNDR-12011-11/2022|EPI_ISL_17065849|2022-01-29</td><td>PX441927</td><td>1/29/2022</td><td>BA.1</td></tr>
<tr><td>hCoV-19/Nicaragua/CNDR-1191-01-SR1/2022</td><td>PX441922</td><td>2/3/2022</td><td>BA.1</td></tr>
<tr><td>hCoV-19/Nicaragua/CNDR-11718-02-SR1/2022</td><td>PX441923</td><td>2/4/2022</td><td>BA.1</td></tr>
<tr><td>hCoV-19/Nicaragua/CNDR-6132-02-SR3/2022</td><td>PX441925</td><td>2/7/2022</td><td>BA.1</td></tr>
<tr><td>hCoV-19/Nicaragua/CNDR-9601-01-SR3/2022</td><td>PX441924</td><td>2/14/2022</td><td>BA.1</td></tr>
<tr><td>hCoV-19/Nicaragua/CNDR-11555-04/2022</td><td>PX441926</td><td>2/17/2022</td><td>BA.1</td></tr>
<tr><td>hCoV-19/Nicaragua/CNDR-13222-01/2022|EPI_ISL_17065836|2022-05-25</td><td>PX441933</td><td>5/25/2022</td><td>BA.2</td></tr>
<tr><td>hCoV-19/Nicaragua/CNDR-12314-08/2022|EPI_ISL_17065833|2022-06-01</td><td>PX441934</td><td>6/1/2022</td><td>BA.2</td></tr>
<tr><td>hCoV-19/Nicaragua/CNDR-10996-05/2022|EPI_ISL_17065832|2022-06-02</td><td>PX441935</td><td>6/2/2022</td><td>BA.2</td></tr>
<tr><td>hCoV-19/Nicaragua/CNDR-7514-18/2022|EPI_ISL_17065838|2022-06-02</td><td>PX441932</td><td>6/2/2022</td><td>BA.2</td></tr>
<tr><td>hCoV-19/Nicaragua/CNDR-13211-01/2022|EPI_ISL_17065835|2022-06-08</td><td>PX441937</td><td>6/8/2022</td><td>BA.2</td></tr>
<tr><td>hCoV-19/Nicaragua/CNDR-9262-17/2022|EPI_ISL_17065839|2022-06-08</td><td>PX441931</td><td>6/8/2022</td><td>BA.2</td></tr>
<tr><td>hCoV-19/Nicaragua/CNDR-10073-03/2022|EPI_ISL_17065831|2022-06-09</td><td>PX441936</td><td>6/9/2022</td><td>BA.2</td></tr>
<tr><td>hCoV-19/Nicaragua/CNDR-11020-01-SR2/2022</td><td>PX441921</td><td>6/20/2022</td><td>BA.2.9</td></tr>
</table>

Supplementary figures

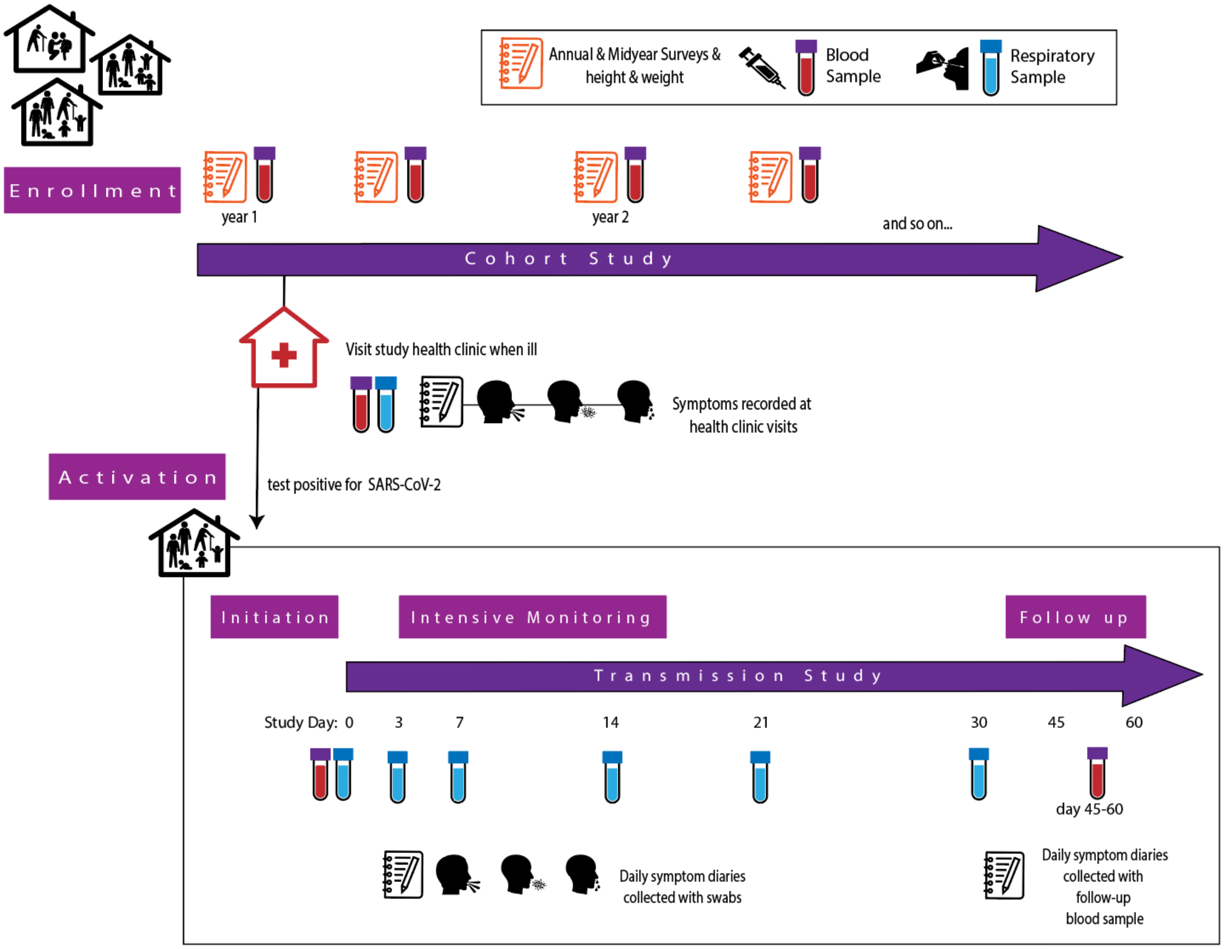

Fig S1. HICS Study design and nested transmission study design for SARS-CoV-2 activations. Adapted from our previously published diagram (Clinical spectrum of Severe Acute Respiratory Syndrome Coronavirus 2 infection and protection from symptomatic reinfection by Maier et al., supplemental study diagram, doi: https://doi.org/10.1093/cid/ciab717), with permission from Oxford University Press. Icons were sourced from The Noun Project under a subscription license that permits publication without attribution.

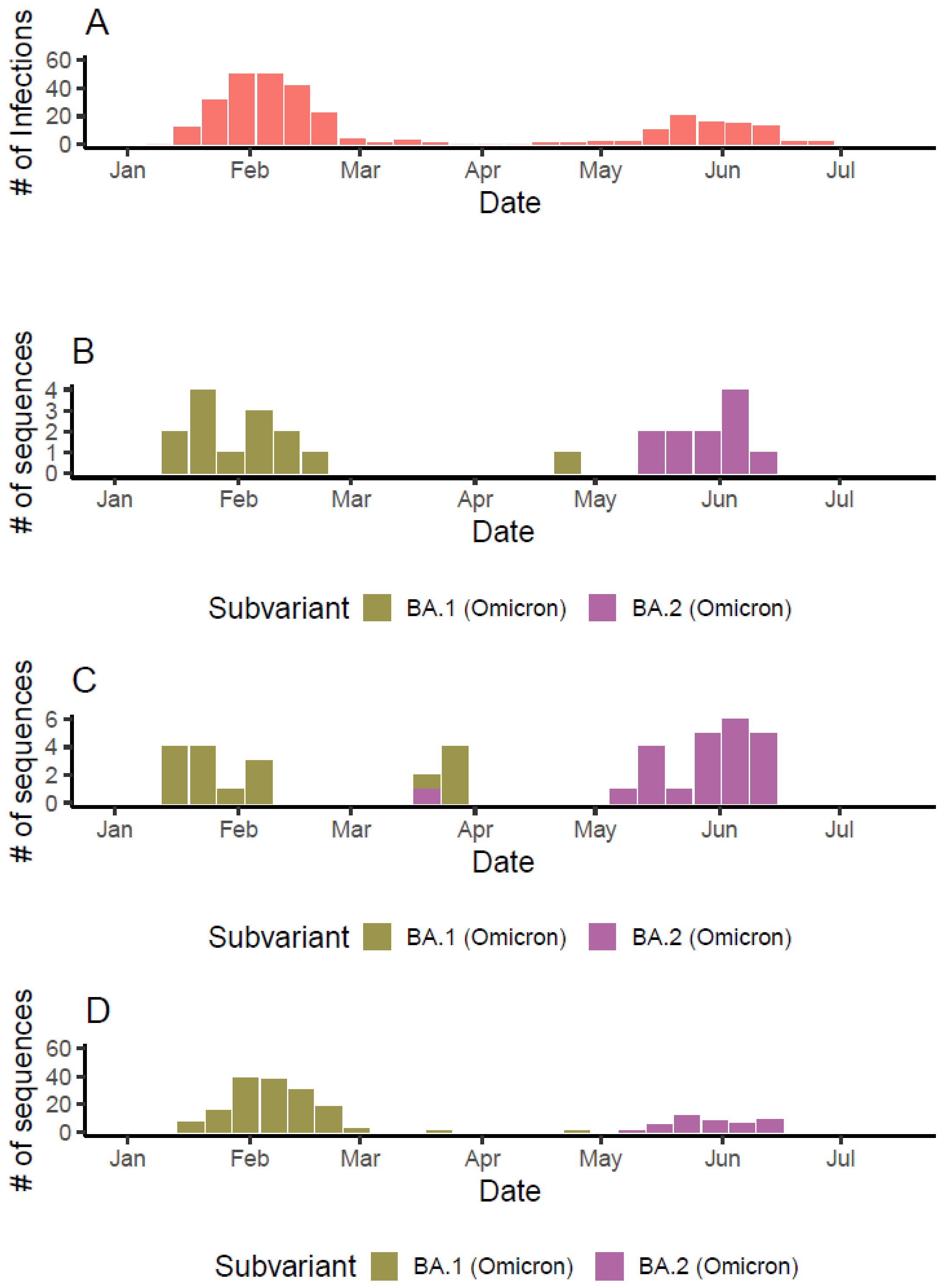

Figure S2. Infections in the HICS study, and sequencing data. (**A**) Total number of cases in the HICS study. (**B**) HICS individuals with sequencing data in the study period. (**C**) Sequenced patients from the Managua Department, extracted from: https://doi.org/10.1101/2024.06.03.596876. (**D**) Final sample with imputed sequencing data.

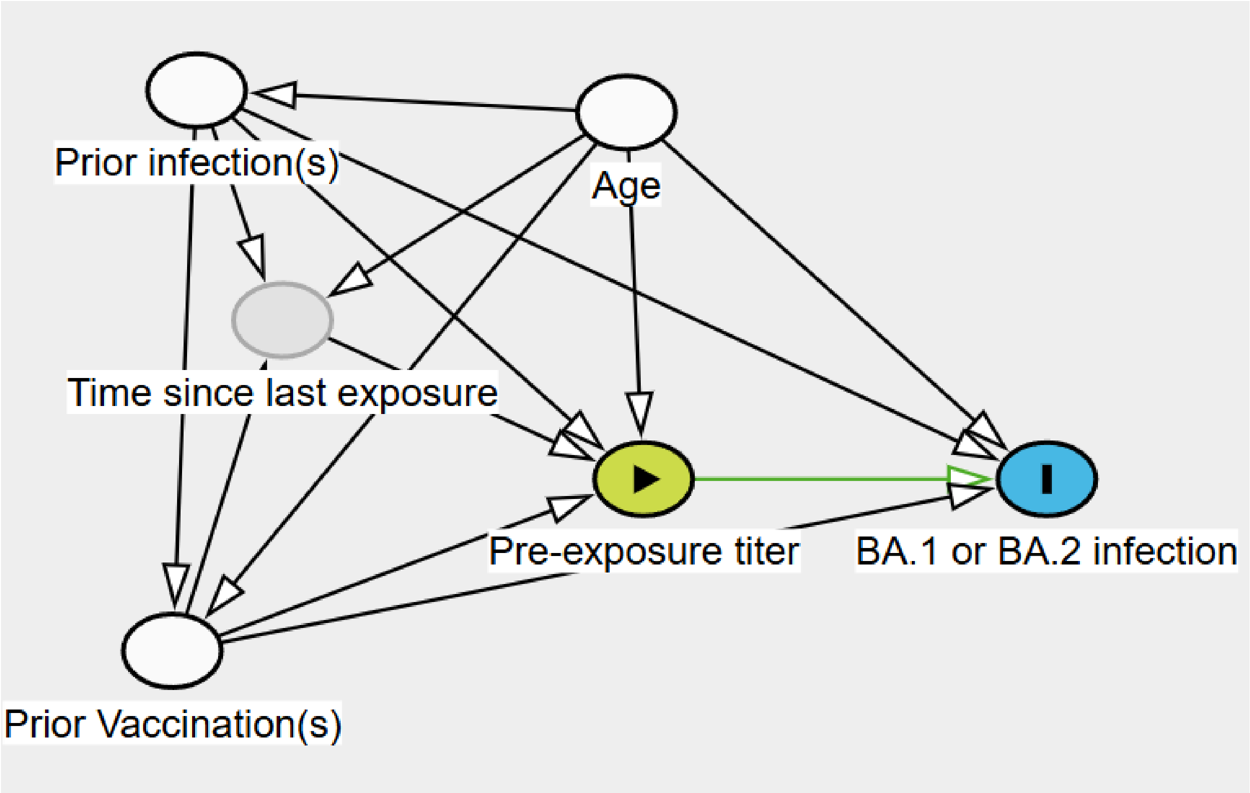

Figure S3. Directed Acyclic Graph analysis. This directed acyclic graph (DAG) illustrates the hypothesized relationships among key variables influencing susceptibility to BA.1 or BA.2 infection. Solid White nodes indicate variables adjusted for the analysis. The yellow node (Pre-exposure titer) is the principal variable of interest. The outcome is the blue node (BA.1 or BA.2 infection). The gray node is an unadjusted covariate. Solid black arrows depict putative causal pathways among variables adjusted for or considered potential confounders. The green arrow from Pre-exposure titer to BA.1 or BA.2 infection highlights the primary unbiased path of interest. Arrows flow from each variable to the factor or outcome it is hypothesized to influence, including between variables adjusted for, such as prior vaccinations and prior infections.

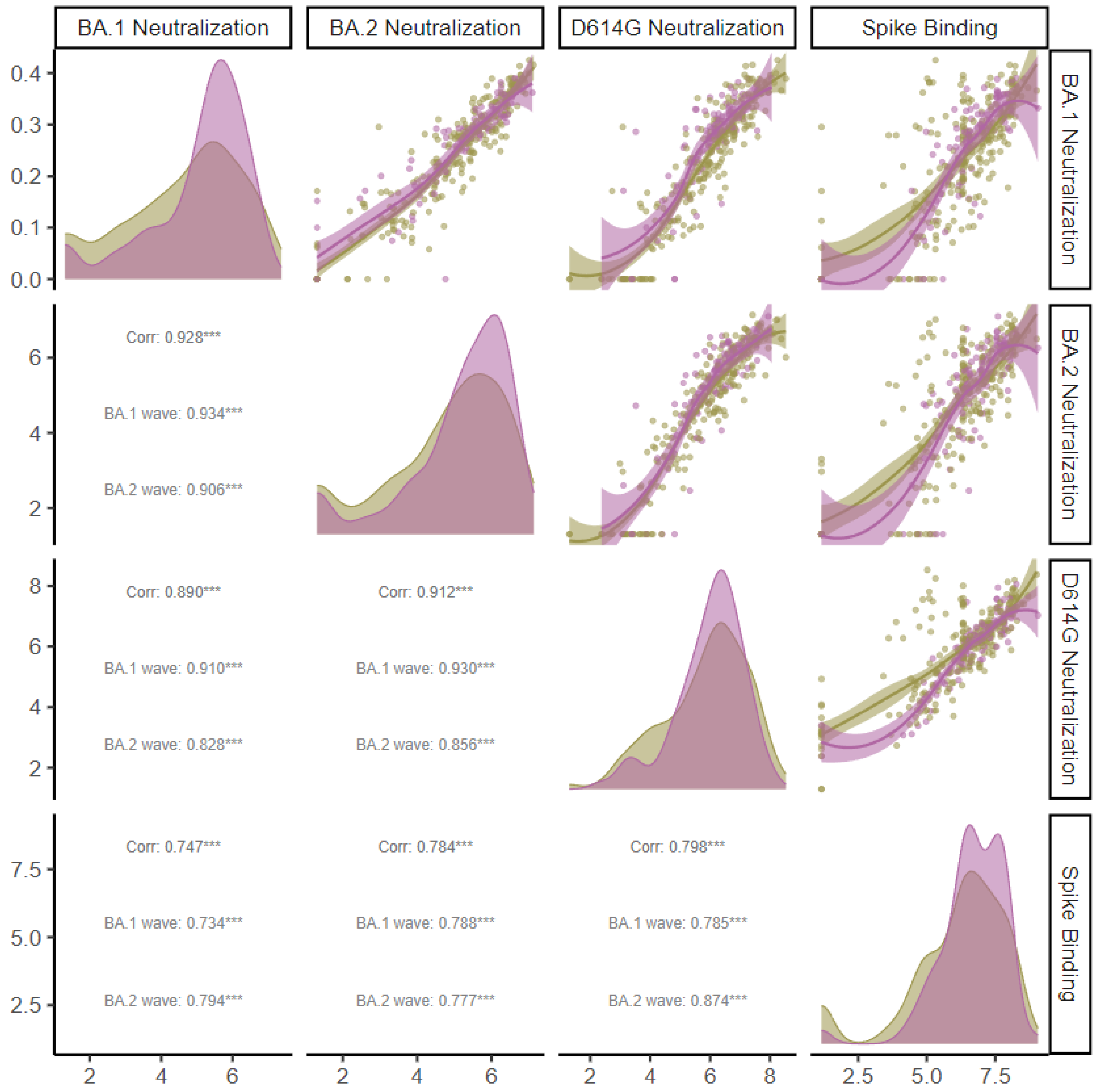

Figure S4. Correlations between assays. Pairwise correlations between BA.1 neutralization, BA.2 neutralization, D614G neutralization, and Spike-binding ELISA across all 345 study participants. Overall Pearson correlations are indicated as "Corr," while wave-specific correlations are labeled as "BA.1 wave" and "BA.2 wave." Correlation coefficients are displayed with levels of statistical significance: ***, **, and * corresponding to P-values of <0.001, <0.010, and <0.050, respectively, with statistical significance assessed via 2-sided tests. The diagonal histograms represent the titer distributions by omicron wave and assay. The distributions for the BA.1 wave are shown in golden-brown, while those for the BA.2 wave are depicted in purple. Each off-diagonal panel visualizes the relationship between two antibody responses, raw values and smooth LOESS correlations along with the 95% CI band colored in gray.

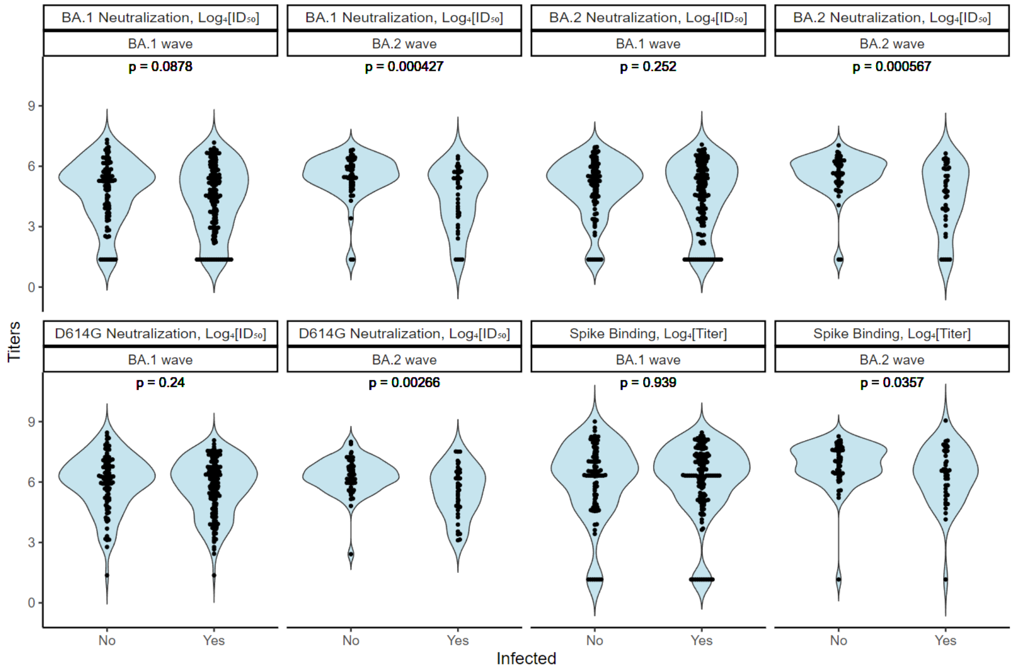

Figure S5. Univariate distributions by infection status. Univariate distributions of antibody responses by infection status during the BA.1 and BA.2 waves across all 345 study participants. P-values were calculated using the Wilcoxon rank-sum test, with statistical significance assessed via 2-sided tests.

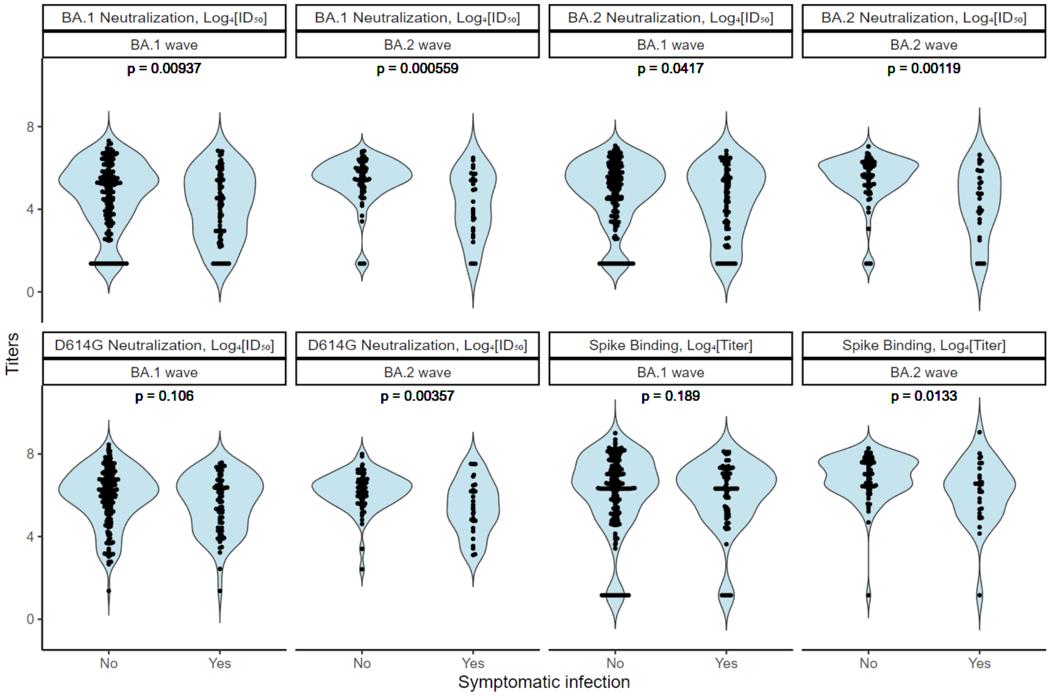

Figure S6. Univariate distributions by symptomatic infection status. Univariate distributions of antibody responses by symptomatic infection status during the BA.1 and BA.2 waves across all 345 study participants. P-values were calculated using the Wilcoxon rank-sum test, with statistical significance assessed via 2-sided tests.

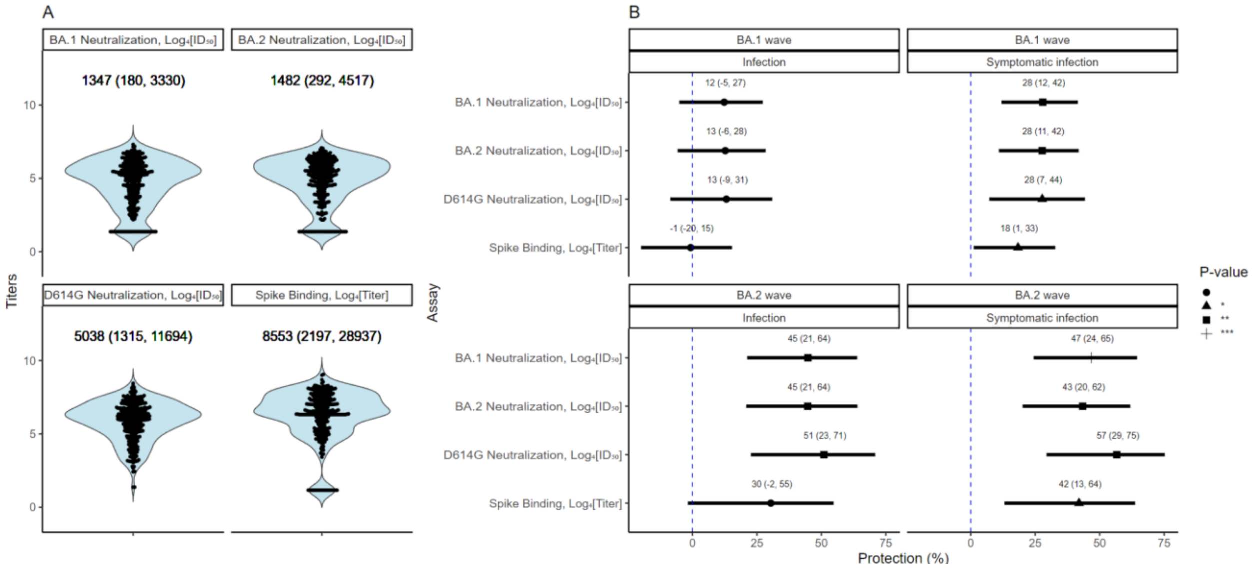

Figure S7. Univariate titer distributions and linear increase in neutralizing and binding titers as protective factors. (**A**) Univariate distributions by assay across Omicron waves and all 345 study particiapants. Untransformed median estimates and 25% and 75% percentiles are shown (**B**)This figure illustrates the effect of every four-fold rise in the likelihood of infection or symptomatic infection. Each estimate (per omicron wave, assay and outcome) is the result of single logistic regression adjusting by age, any prior infection, and any prior vaccination, with statistical significance assessed via 2-sided Wald tests. Odds ratio estimates were converted into protection estimates expressed as percentages ([1-OR] x 100). Dashed line indicates null effect. P-values are indicated for statistical significance, with ***, , and * corresponding to P-values of <0.001, <0.010, and <0.050, respectively. No adjustments for multiple comparisons were applied. Exact P-values are shown in Tables S3 and S4.

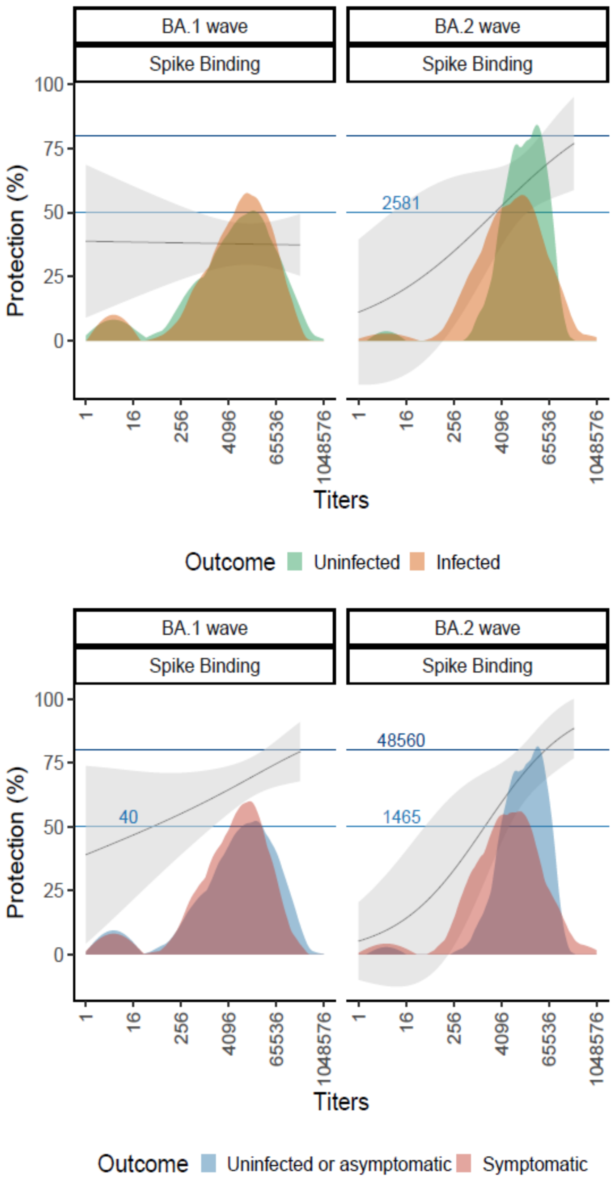

Figure S8. Protection curves for binding assays by Omicron wave and assay. This figure illustrates the relationship between binding titers and protection against BA.1 and BA.2 infection and symptomatic infection during their respective waves. The solid lines represent the mean predicted protection curves, calculated as 1 - predicted risk, according to a Generalized Additive Model using a Gaussian distribution (see Methods), with the shaded ribbon bands denoting the 95% confidence intervals (CIs) around the mean predicted protection estimates, derived from vaccinated and prior-infected adults. The vertical dashed lines mark the points where the predicted protection curves cross the 50% and 80% protection thresholds, highlighting the titer levels required to achieve these levels of protection. Embedded within the figure are histograms showing the densities of neutralization titers, stratified by outcome (infection vs. no infection, symptomatic vs. asymptomatic). Data were derived from all 345 study participants stratified by Omicron wave and outcome: BA.1 wave (n = 251; 143 infected vs. 108 uninfected, 105 symptomatic vs. 146 not symptomatic) and BA.2 wave (n = 94; 41 infected vs. 53 uninfected, 29 symptomatic vs. 65 not symptomatic), with one pre-exposure sample per participant. Binding titers were obtained by averaging replicate measurements across technical triplicates of each sample.
