# Supplementary material for: Variant-specific antibody correlates of protection against SARS-CoV-2 Omicron symptomatic and overall infections
Source: Nat Commun. 2025 Nov 21;16:10305. doi: 10.1038/s41467-025-65235-8 (PMC12639126; doi:10.1038/s41467-025-65235-8)
Supplement: Supplementary file 2 — Reporting Summary [file 41467_2025_65235_MOESM2_ESM.pdf]

nature portfolio

Corresponding author(s): Aubree Gordon, David D. Ho

Last updated by author(s): Sep 14, 2025

Reporting Summary

Nature Portfolio wishes to improve the reproducibility of the work that we publish. This form provides structure for consistency and transparency in reporting. For further information on Nature Portfolio policies, see our Editorial Policies and the Editorial Policy Checklist.

Statistics

For all statistical analyses, confirm that the following items are present in the figure legend, table legend, main text, or Methods section.

n/a	Confirmed	
☐	☒	The exact sample size (*n*) for each experimental group/condition, given as a discrete number and unit of measurement
☐	☒	A statement on whether measurements were taken from distinct samples or whether the same sample was measured repeatedly
☐	☒	The statistical test(s) used AND whether they are one- or two-sided<br>*Only common tests should be described solely by name; describe more complex techniques in the Methods section.*
☐	☒	A description of all covariates tested
☐	☒	A description of any assumptions or corrections, such as tests of normality and adjustment for multiple comparisons
☐	☒	A full description of the statistical parameters including central tendency (e.g. means) or other basic estimates (e.g. regression coefficient) AND variation (e.g. standard deviation) or associated estimates of uncertainty (e.g. confidence intervals)
☐	☒	For null hypothesis testing, the test statistic (e.g. *F*, *t*, *r*) with confidence intervals, effect sizes, degrees of freedom and *P* value noted<br>*Give P values as exact values whenever suitable.*
☒	☐	For Bayesian analysis, information on the choice of priors and Markov chain Monte Carlo settings
☒	☐	For hierarchical and complex designs, identification of the appropriate level for tests and full reporting of outcomes
☒	☐	Estimates of effect sizes (e.g. Cohen's *d*, Pearson's *r*), indicating how they were calculated

Our web collection on statistics for biologists contains articles on many of the points above.

Software and code

Policy information about availability of computer code

Data collection	Neutralization data were collected using the following softwares: SoftMax Pro v.7.0.2 (Molecular Devices). Demographic, vaccine and ELISA data were collected using HeidiSQL v12.11 or Microsoft Access (Access 2024). Infection histories were determined using SAS software v9.4.
Data analysis	R Studio v4.3.1 was utilized for data analysis no custom algorithm was used. Code link is available on the manuscript: https://zenodo.org/records/16804602

For manuscripts utilizing custom algorithms or software that are central to the research but not yet described in published literature, software must be made available to editors and reviewers. We strongly encourage code deposition in a community repository (e.g. GitHub). See the Nature Portfolio guidelines for submitting code & software for further information.

Data

Policy information about availability of data

All manuscripts must include a data availability statement. This statement should provide the following information, where applicable:

- Accession codes, unique identifiers, or web links for publicly available datasets
- A description of any restrictions on data availability
- For clinical datasets or third party data, please ensure that the statement adheres to our policy

Researchers interested in accessing the study data are encouraged to submit a formal request to A.G. or the Committee for the Protection of Human Subjects at the University of Michigan. To uphold ethical standards and ensure appropriate data use, each request will undergo a case-by-case review and approval process.

Additionally, as the data include information collected in Nicaragua, access is subject to Nicaraguan data ownership regulations and may require approval from relevant Nicaraguan authorities. Materials requests and correspondence should be directed to A.G. (gordonal@umich.edu) and D.D.H. (dh2994@cumc.columbia.edu).

Research involving human participants, their data, or biological material

Policy information about studies with human participants or human data. See also policy information about sex, gender (identity/presentation), and sexual orientation and race, ethnicity and racism.

Reporting on sex and gender	Self-reported biological sex was used as confounder variable. Biological sex was not confirmed by genetic testing. Findings apply to both sexes.
Reporting on race, ethnicity, or other socially relevant groupings	No variables utilizing race, ethnicity, or other socially relevant categorization were used in this manuscript for purposes of study design. This study includes both genders and all races. No potential participant in the cohort study was excluded based on race. The racial composition of the cohort is representative of the source population.
Population characteristics	All participants live in District II, Managua, Nicaragua, the catchment area for the Health Center Socrates Flores Vivas (HCSFV). The age of study participants was limited to individuals ages 0 and up. Population has a low-to-middle income socioeconomic status (SES). District II is an urban area.
Recruitment	Families were recruited from current pediatric influenza cohort participants. This enabled the study team to access extensive infection histories and samples dating back up to 7 years for some participants. 395 Households from these children were selected randomly to participate in the household cohort.<br><br>Inclusion criteria: For inclusion in the study, a participant must (1) live in the HCSFV catchment area, (2) live in a household with at least 1 child aged 12 or under, (3) intend to remain in the neighborhood for the length of the study; (4) have a vaccination card (children) or ID document (adults) in order to confirm age and residence, or, if a vaccination card cannot be presented, children must have a pre-existing medical chart at the HCSFV; and (5) if a participant is sick (acute and/or chronic illness), he/she must be examined at the HCSFV to determine eligibility for participation in the study. No exclusion criteria.
Ethics oversight	These studies received approval from the institutional review boards of the Nicaraguan Ministry of Health and the University of Michigan (HUM00119145 and HUM00178355). Written informed consent or parental permission was obtained for all enrolled participants, and children aged 6 years or older also provided assent.

Note that full information on the approval of the study protocol must also be provided in the manuscript.

Field-specific reporting

Please select the one below that is the best fit for your research. If you are not sure, read the appropriate sections before making your selection.

☒ Life sciences ☐ Behavioural & social sciences ☐ Ecological, evolutionary & environmental sciences

For a reference copy of the document with all sections, see nature.com/documents/nr-reporting-summary-flat.pdf

Life sciences study design

All studies must disclose on these points even when the disclosure is negative.

Sample size	Households selected in this study experienced the introduction of BA.1 or BA.2 SARS-CoV-2 strains by a household member, with these strains circulating consecutively between January and June 2022. All activated households within these period were selected except for two households because they lacked sufficient data for direct sequencing or single imputation (multiple strains were circulating that week). Final sample size is 345 participants, 251 within the BA.1 Omicron wave and 94 individuals within the BA.2 Omicron wave.
Data exclusions	Two households (totaling 11 individuals) were subsequently excluded because they lacked sufficient data for direct sequencing or single imputation (multiple strains were circulating that week).
Replication	Neutralization experiments were replicated on different days using samples from the cohort. Within each experimental replicate, there were technical triplicates for each combination of serum sample, pseudovirus, and serum dilution. One dilution Spike ELISAs were run in duplicates while titration data was duplicated randomly on 20% of all plates.
Randomization	No randomization was performed.
Blinding	Blinding was not relevant to our study. Controlled confounders in our study included age (continuous variable), vaccination status (any prior vaccination, dichotomous variable) and infection history (any prior infection, dichotomous variable). These were selected based on Directed Acyclic Graph analysis and literature review. These were controlled for by using multivariate regressions.

Reporting for specific materials, systems and methods

We require information from authors about some types of materials, experimental systems and methods used in many studies. Here, indicate whether each material, system or method listed is relevant to your study. If you are not sure if a list item applies to your research, read the appropriate section before selecting a response.

Materials & experimental systems

n/a	Involved in the study
☒	☐ Antibodies
☐	☒ Eukaryotic cell lines
☒	☐ Palaeontology and archaeology
☒	☐ Animals and other organisms
☐	☒ Clinical data
☒	☐ Dual use research of concern
☒	☐ Plants

Methods

n/a	Involved in the study
☒	☐ ChIP-seq
☒	☐ Flow cytometry
☒	☐ MRI-based neuroimaging

Eukaryotic cell lines

Policy information about cell lines and Sex and Gender in Research

Cell line source(s)	Vero-E6 (CRL-1586) cells and HEK293T (CRL-3216) cells were obtained from ATCC
Authentication	These cell lines were provided by the original manufacturer. Cell morphology was assessed before each experiment.
Mycoplasma contamination	All cells were assessed for mycoplasma contamination using Lonza MycoAlert kits.
Commonly misidentified lines (See ICLAC register)	Cell line morphology of HEK293T and Vero-E6 was visually confirmed with each passage. Neither cell line is listed in the ICLAC database of misidentified or cross-contaminated cell lines (https://iclac.org/databases/cross-contaminations/).

Clinical data

Policy information about clinical studies

All manuscripts should comply with the ICMJE guidelines for publication of clinical research and a completed CONSORT checklist must be included with all submissions.

Clinical trial registration

Protocols HUM00119145 and HUM00178355

Study protocol

Study Protocols are available by request, as is required by the Institutional Review Board-approved protocol for the Dynamics of Influenza Transmission in Nicaraguan Households. Please contact Dr. Aubree Gordon and/or Health Sciences and Behavioral Sciences Institutional Review Board (IRB-HSBS) at University of Michigan

Data collection

The Household Influenza Cohort Study (HICS) is an ongoing prospective cohort study in District II of Managua, Nicaragua in the Health Center Socrates Flores Vivas (HCSFV). Established in 2017 to investigate influenza, it was expanded in early 2020 to include SARS-CoV-2 infection and disease. All cohort participants undergo regular blood sampling—twice per year in 2020 and 2021, and then annually thereafter. At the first sign of any illness, participants are instructed to seek care at the study health clinic, where they receive primary care and diagnostic testing.

An embedded household transmission study is activated once a participating household member is confirmed positive for SARS-CoV-2. After activation, study personnel visit the household on days 0, 3, 7, 14, 21, and 30 to collect combined nasal/oropharyngeal swabs, maintain symptom diaries, and test all household members regardless of symptoms. Serum samples are collected on the same day as activation. Active surveillance of household contacts allows the detection of symptomatic and asymptomatic SARS-CoV-2 infections.

Clinical consultations at the health center and in homes are used to collect symptom data. Daily symptom diaries are maintained during intensive monitoring for COVID-positive households. Trained staff enter clinical and symptom data into mobile devices, which sync to a secure, quality-controlled database. QC procedures are in place for all data collection and lab analysis. Data undergo monthly checks and are validated for consistency. Identifiable information is stored separately, encrypted, and access is restricted to essential staff.

Blood samples were analyzed in pairs (current vs. baseline) using enzyme-linked immunosorbent assay (ELISA) protocols adapted from the Krammer laboratory for the detection of antibodies against SARS-CoV-2 spike receptor binding domain (RBD), Spike and Nucleoprotein (NP). The RBD and spike proteins used in these assays were produced in single batches at the Life Sciences Institute at the University of Michigan, based on the original SARS-CoV-2 strain. Due to its specificity, RBD was used for initial screening (positive/negative), while positive samples were subsequently titrated using the spike assay. The NP ELISA was also performed to differentiate vaccine-induced responses from those due to infection. Real-time reverse-transcription polymerase chain reaction (RT-PCR) was conducted following the protocol described by Chu et al.17

Neutralization titers against BA.1, BA.2 and D614G SARS-CoV-2 strains were measured using vesicular stomatitis virus–based pseudoviruses (VSV) bearing different SARS-CoV-2 spike proteins produced in HEK293T cells, as previously described.11 After transfecting spike-encoding plasmids, a VSV-G pseudotyped ΔG-luciferase was used to generate the pseudoviruses, which were harvested and standardized by determining the 50% tissue culture infectious dose (TCID50) in Vero-E6 cells. Heat-inactivated serum samples were then serially diluted, incubated with each pseudovirus, and added to Vero-E6 cells, followed by a 16-hour incubation.

Luciferase activity was subsequently measured, and ID50 values for each strain-specific pseudovirus were calculated using a five-parameter log-logistic model to compare neutralization responses across the different SARS-CoV-2 variants.

Outcomes

Symptoms reported in daily symptom diaries and during clinic visits were used to categorize illness severity into four levels. “Severe” cases included individuals requiring hospitalization, and those deemed in need of hospitalization by clinicians. No deaths due to COVID-19 were recorded during the Omicron BA.1 or BA.2 waves in the HICS study. “Moderate” cases encompassed individuals presenting with difficulty breathing, shortness of breath, rapid breathing, tight chest feeling, chest pain, ALRI, SARI, crepitus, chest wall indrawing, rhonchi, wheezing, or an overall poor condition. “Mild” cases were defined as those with loss of smell/taste, fever, or at least two other symptoms. Finally, “subclinical” cases involved no more than one symptom that did not meet the criteria for higher severity. The primary endpoints of this study were the percentage protection against RT-PCR-confirmed infection—including subclinical, mild, moderate, and severe infections, (hereafter referred to as ‘infection’)—and symptomatic infection (including mild, moderate, and severe infections) among households with SARS-CoV-2 BA.1 or BA.2 exposure.

This study is subject to several potential sources of bias that could affect the interpretation of results. Ascertainment bias may arise from the household-based design, as households with greater susceptibility to transmission and disease were more likely to be enrolled, potentially limiting generalizability. Variability in neutralization assay methodologies across laboratories could influence the exact protective thresholds reported, while limited sample size may reduce power to detect correlates of protection, particularly for BA.1. Additional concerns include possible unreported infections between waves, assumptions of equal exposure within households, and unmeasured confounders such as health-seeking behavior, comorbidities, genetics, and socioeconomic status, all of which, along with violations of mediation assumptions, may bias estimates and limit causal interpretation.

Plants

Seed stocks

Report on the source of all seed stocks or other plant material used. If applicable, state the seed stock centre and catalogue number. If plant specimens were collected from the field, describe the collection location, date and sampling procedures.

Novel plant genotypes

Describe the methods by which all novel plant genotypes were produced. This includes those generated by transgenic approaches, gene editing, chemical/radiation-based mutagenesis and hybridization. For transgenic lines, describe the transformation method, the number of independent lines analyzed and the generation upon which experiments were performed. For gene-edited lines, describe the editor used, the endogenous sequence targeted for editing, the targeting guide RNA sequence (if applicable) and how the editor was applied.

Authentication

Describe any authentication procedures for each seed stock used or novel genotype generated. Describe any experiments used to assess the effect of a mutation and, where applicable, how potential secondary effects (e.g. second site T-DNA insertions, mosiacism, off-target gene editing) were examined.
